# Supplementary material for: The little skate genome and the evolutionary emergence of wing-like fins
Source: Nature. 2023 Apr 12;616(7957):495–503. doi: 10.1038/s41586-023-05868-1 (PMC10115646; doi:10.1038/s41586-023-05868-1)
Supplement: Supplementary file 1 — This file contains Supplementary Figs. 1–16 and a guide to the Supplementary Tables. [file 41586_2023_5868_MOESM1_ESM.pdf]

nature portfolio

https://doi.org/10.1038/s41586-023-05868-1

Supplementary information

The little skate genome and the evolutionary emergence of wing-like fins

In the format provided by the authors and unedited

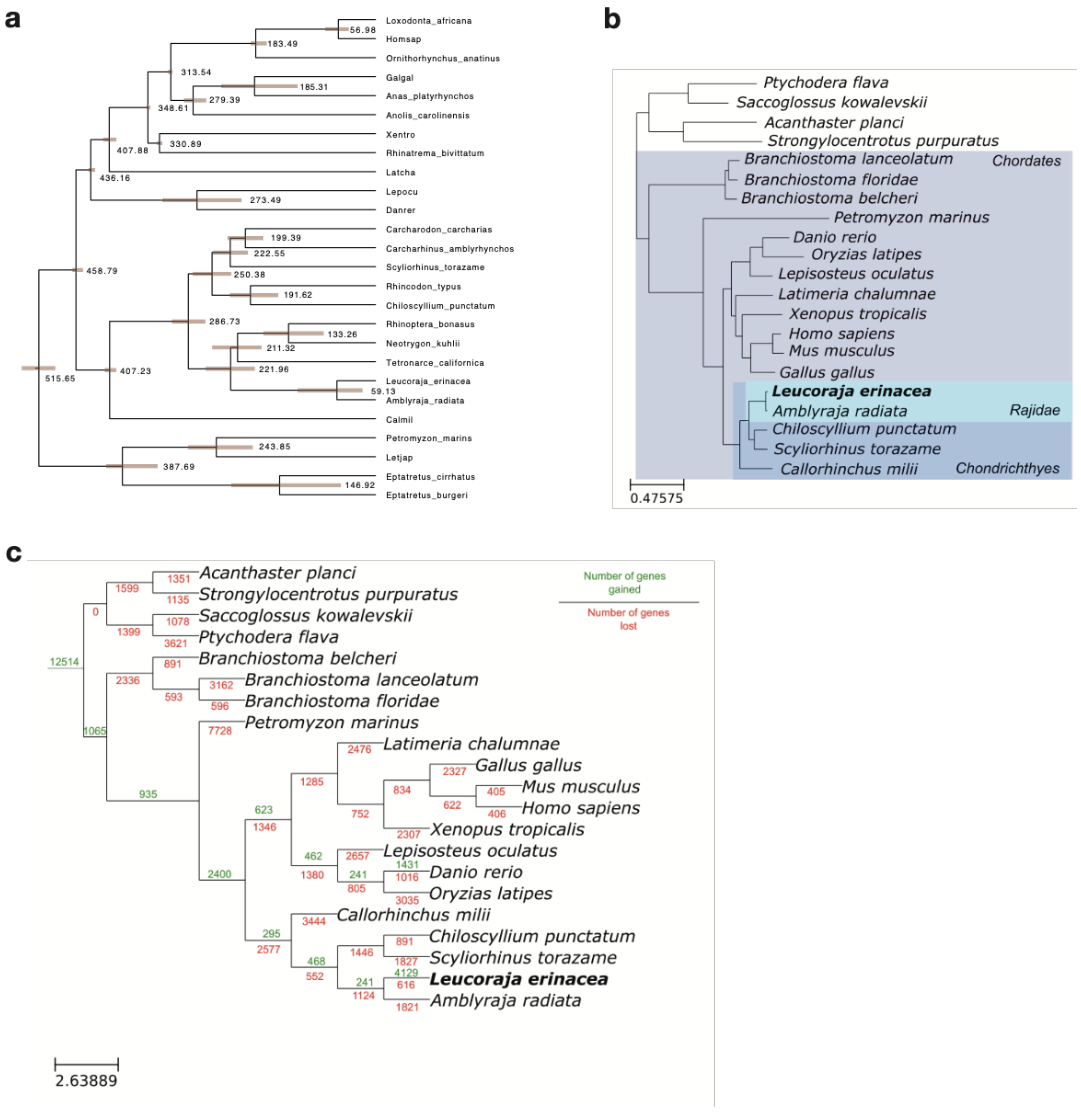

Supplementary Information Figure 1. Extended chronogram and gene gains and loss from Phylome. a, Chronogram of selected vertebrate species based on 74145 aminoacid position analyzed using Phylobayes assuming the CIR model with birth-death priors and and soft-bounds on divergence times (calibrations from [1]). **b**, Species using for Phylome reconstruction and **c,** Gain and loss of genes along the branch as inferred using the Phylome pipeline.

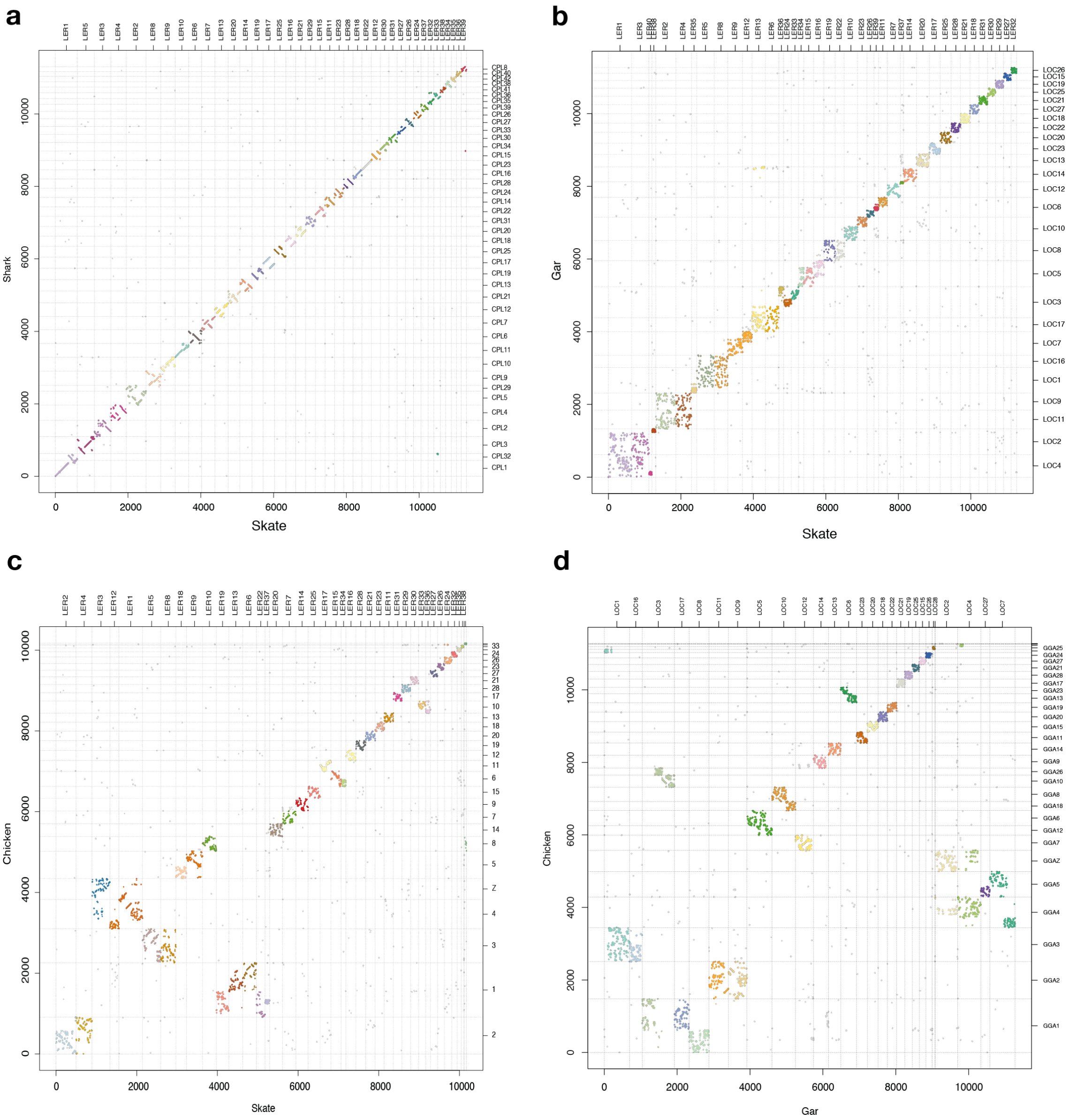

Supplementary Information Figure 2. Synteny and disruption of gene order. a-c, Synteny plots between the little skate (*Leucoraja erinacea*) and selected vertebrate species: **a,** white–spotted bamboo shark (*Chiloscyllium plagiosum*); **b**, gar (*Lepisosteus oculatus*); **c**, chicken (*Gallus gallus*). **d**, Synteny plot between gar and chicken.

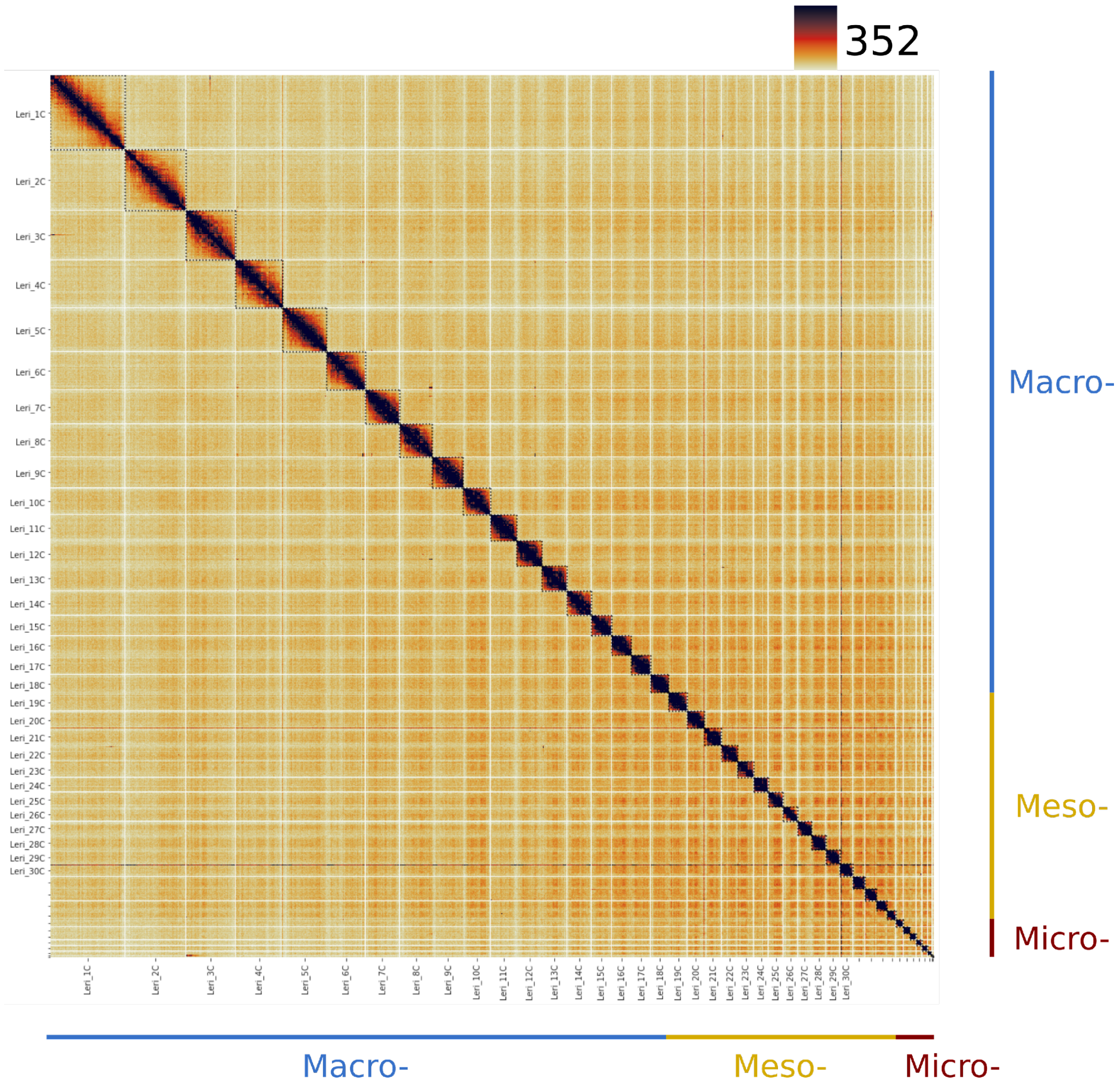

Supplementary Information Figure 3: Skate chromosome territories. Skates display a full set of condensins and therefore their genome is organized in chromosome territories. Hi-C map at 1Mb resolution of the entire *L. erinacea* genome. Chromosome ends are delineated with dashed lines. Intrachromosomal contacts are enriched. Note the increased interchromosomal interaction between microchromosomes.

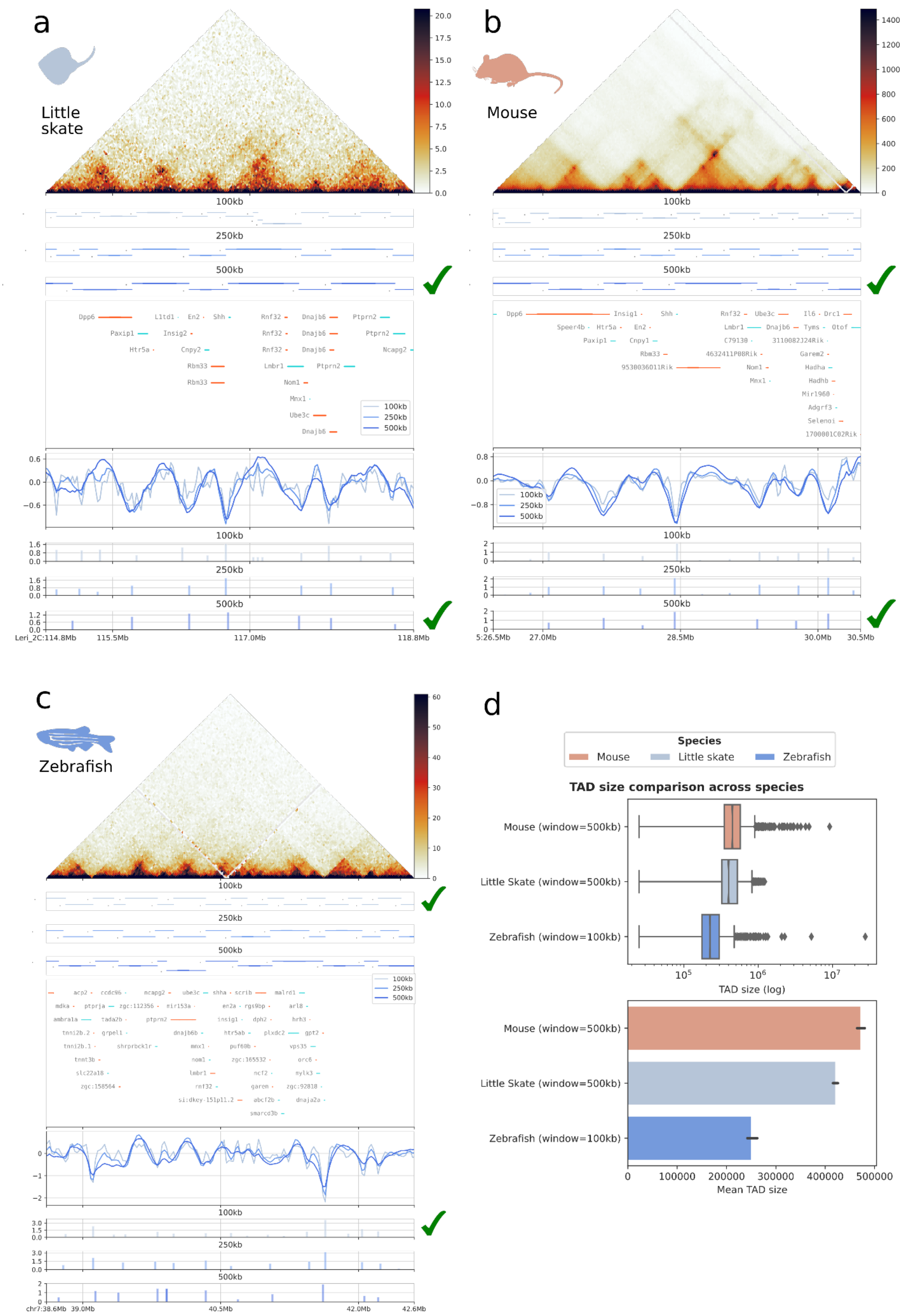

Supplementary Information Figure 4. Skate TADs comparison with other vertebrates. Insulation scores and TAD prediction with different window size parameters (100, 250 and 500kb) in skate embryonic fins (**a**), mouse cortical neurons[2] (**b**), and zebrafish embryos (**c**)[3]. Chosen parameters of insulation score window size used for TAD identification are highlighted with a green tick. **d.** Distribution of TAD

sizes (top) and mean TAD size (bottom) in skate, mouse and zebrafish with chosen window size parameters (n = 5384 TADs in mouse, 4664 TADs in skate and 5304 TADs in zebrafish). Boxes at the top correspond to the median and the first and third quartiles (Q1 and Q3). Whiskers extend to the last point within 1.5 times the interquartile range below and above Q1 and Q3, respectively. Error bars from the barplots at the bottom represent the 95% confidence interval of the mean calculated via bootstrapping (seaborn barplot default).

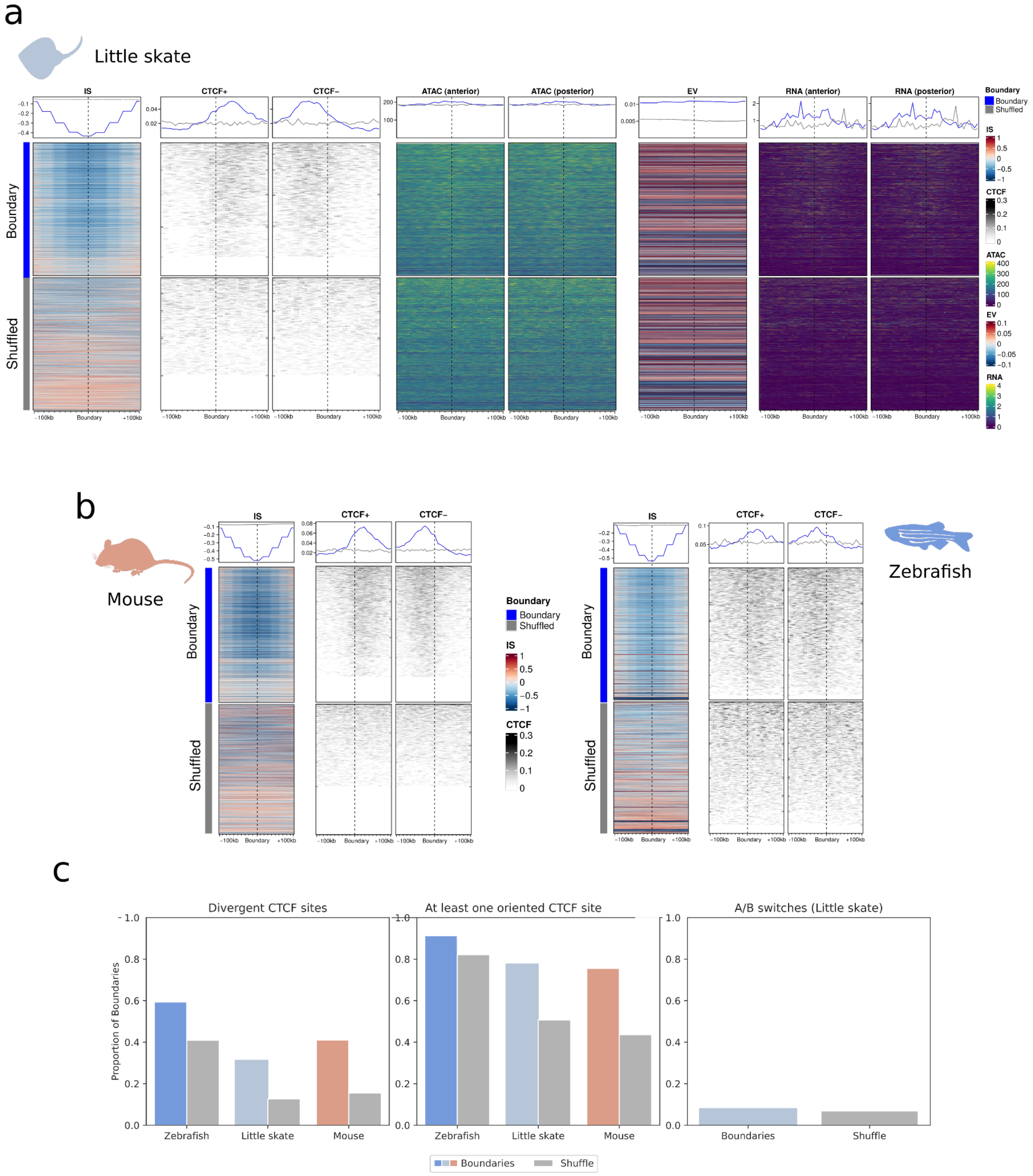

Supplementary Information Figure 5. Features of vertebrate boundaries. a. Insulation score, directional CTCF motifs inside ATAC peaks, ATAC, eigenvector and RNA-seq signals 100kb around TAD boundaries and shuffled sequences in skate. Divergent CTCF motif signature is the most prominent feature of skate boundaries. **b.** Insulation score and CTCF motifs inside CTCF ChIP-seq peaks 100kb around TAD boundaries of mouse cortical neurons and zebrafish embryos (datasets from Supplementary Information Figure 4). **c.** Proportion of boundaries containing divergent CTCF motifs (left) or at least one directional CTCF motif (center) around skate, mouse and zebrafish boundaries in comparison with shuffled controls. Proportion of boundaries with A/B compartment switches in skate.

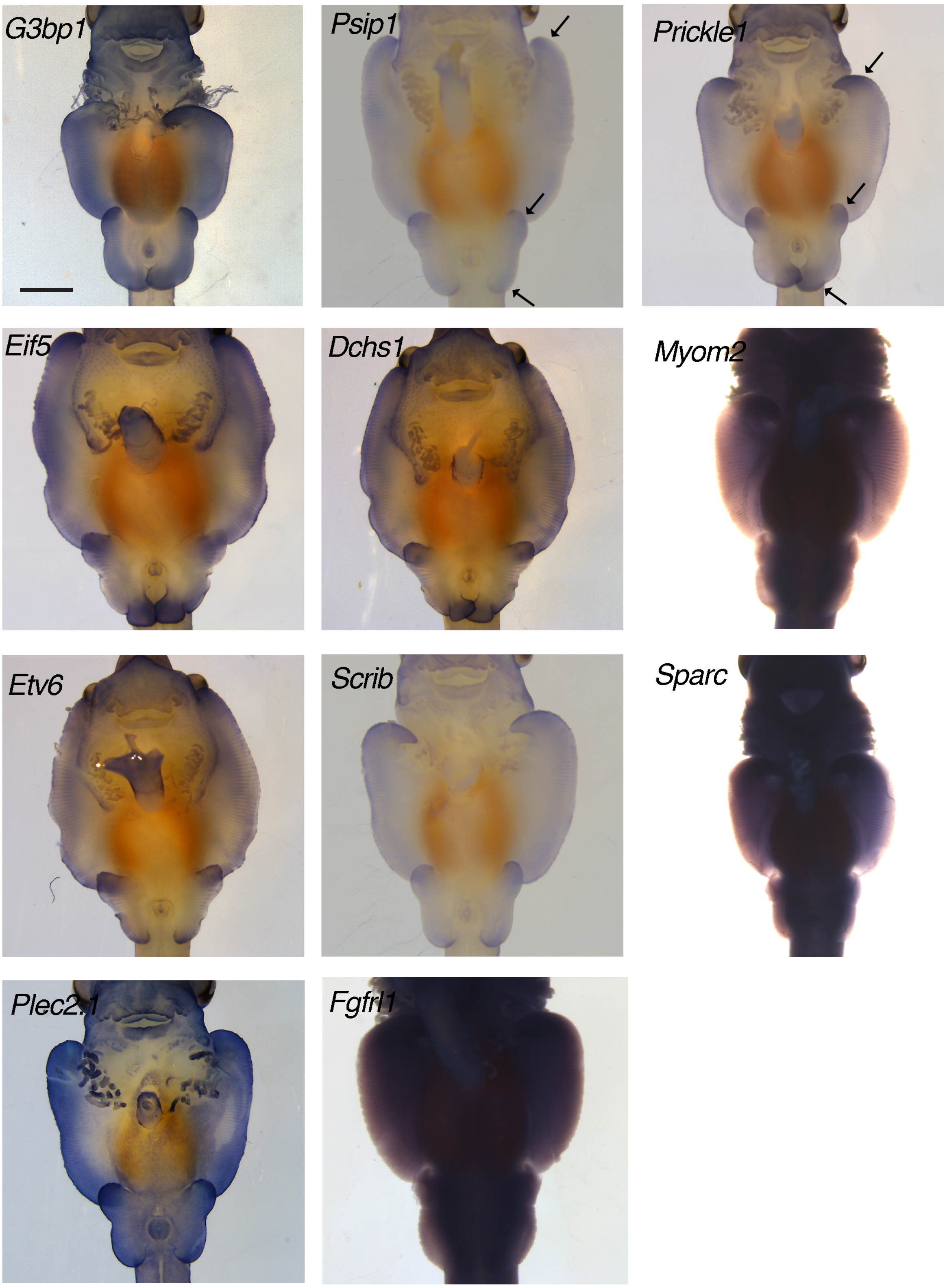

Supplementary Information Figure 6: Whole mount *in situ* hybridization of the selected genes. The expression patterns of the top eight candidate genes for the anterior expansion of the pectoral fin were investigated at stage 31 or 32 by whole mount *in situ* hybridization. While *G3bp1, Eif5, Etv6, Plec2.1, Dchs1, Scrib, Fgfrl1, Myom2,* and *Sparc* showed broad expression patterns in the pectoral fin, *Psip1* and *Prickle1* exhibited the expression in the anterior pectoral and pelvic fins as well as the clasper expression (arrows). $n = 6$ for each gene. The scale bar is 2 mm.

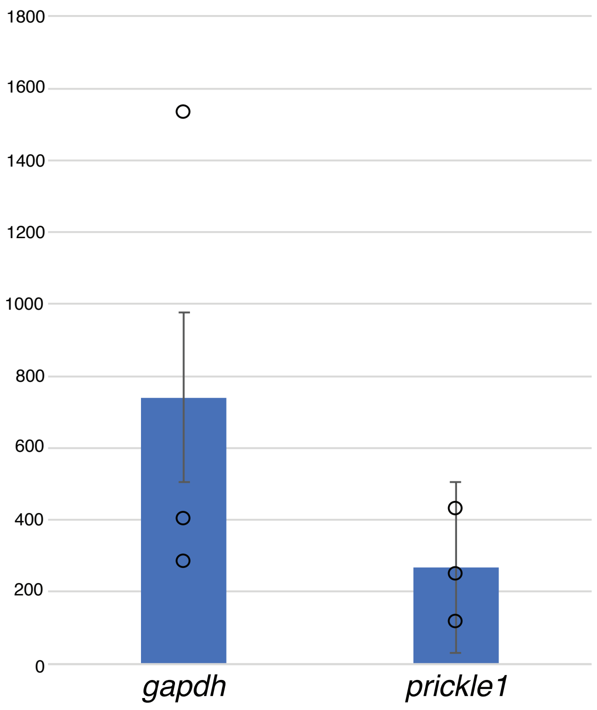

Supplementary Information Figure 7. Real-time PCR of *gapdh* and *prickle 1* in shark pectoral fins. The transcripts of *gapdh* and *prickle1* in the shark pectoral fin (*Scyliorhinus retifer*) at stage 30 were detected by real-time PCR. Three replicates were performed. The vertical axis shows the arbitrary expression values calculated from Ct-values of the real-time PCR. The error bars are standard errors. Both *gapdh* and *prickle 1* transcripts were successfully detected.

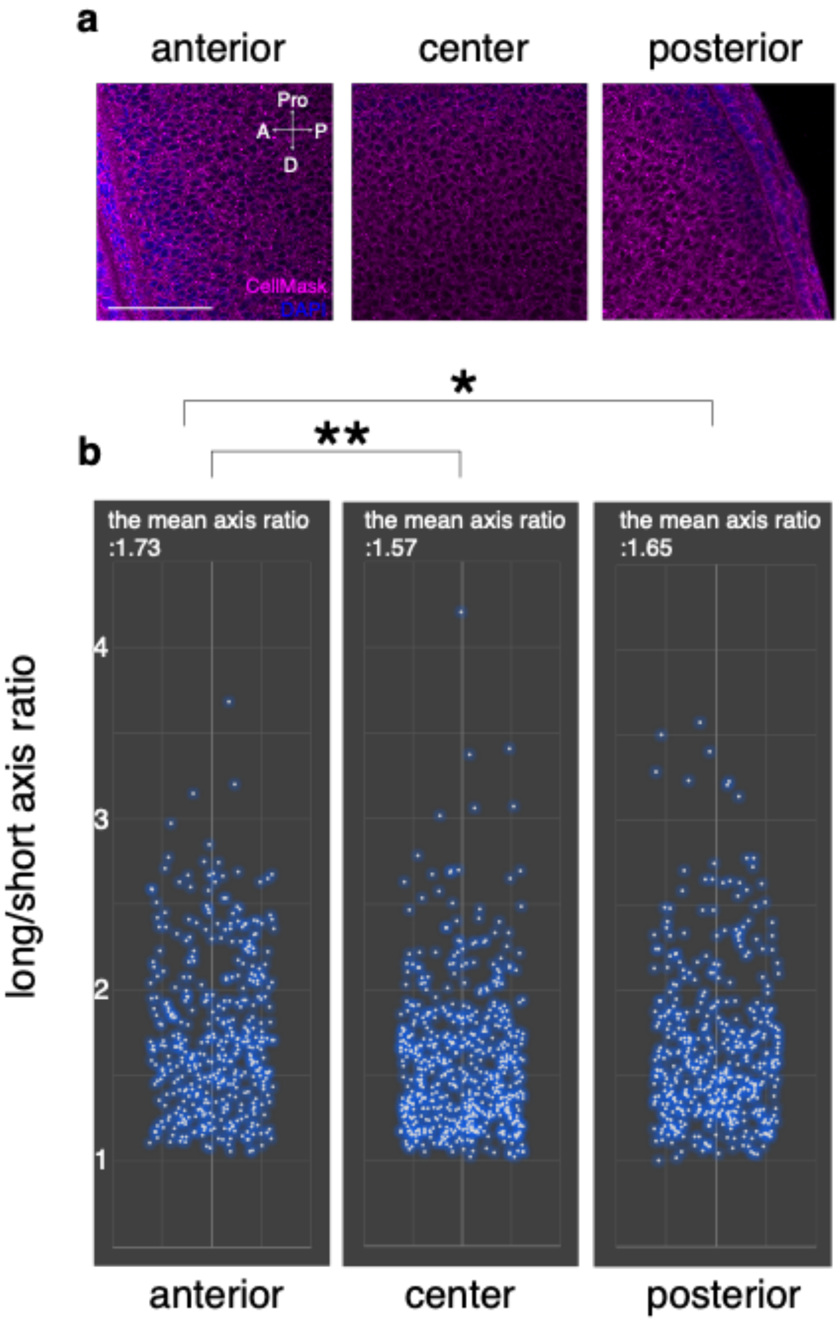

Supplementary Information Figure 8. Cell elongation analysis for skate pectoral fins. a. the representative images of membrane (CellMask) and nucleus (DAPI) staining in the anterior, center, and posterior fins. A;anterior, P; posterior, Pro; proximal, D; distal. **b.** The ratio of the long/short axis length of mesenchymal cells in the anterior, center, and posterior pectoral fins was measured and calculated by Fiji (see the Method) and mapped on the graphs. The anterior cells are more oval compared to center and posterior cells. *p* value of t-test between the anterior and center samples is 2.79 x 10^-06 (**) and between the anterior and posterior samples is 0.0195 (*). The numbers of cells investigated in the anterior, center, and posterior fins are 409, 549, and 431, respectively. The scale bar is 100 μm.

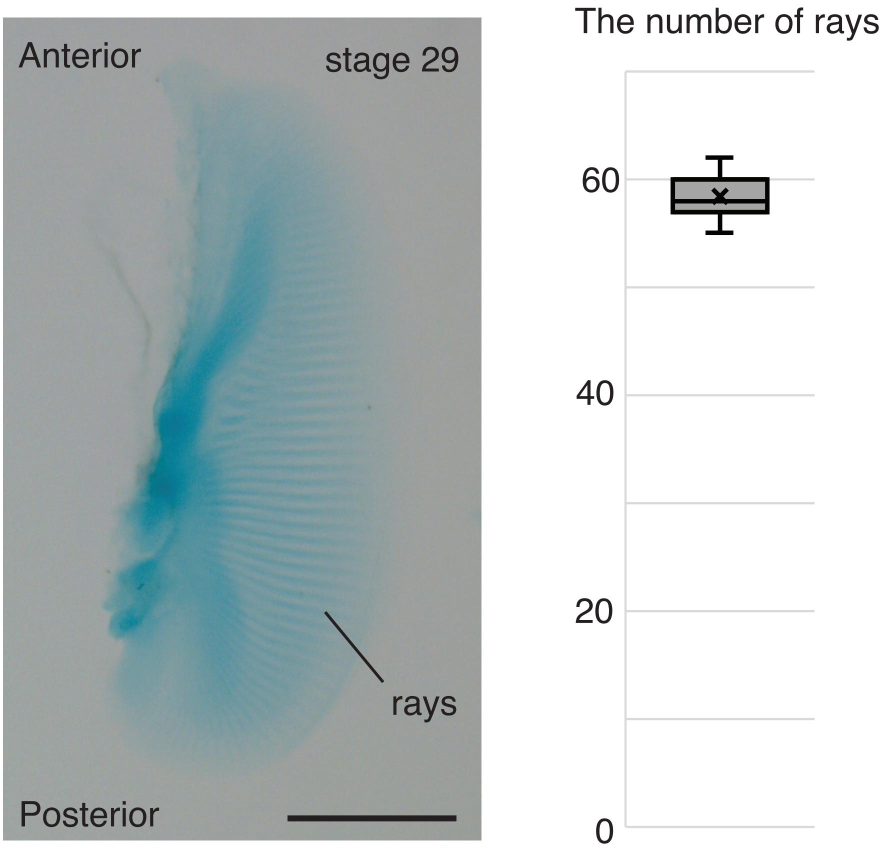

Supplementary Information Figure 9. Cartilage staining of stage 29 pectoral fin. At stage 29, tribasal bones (pro-, meso-, and meta-pterygium) do not show clear distinction among them. Fin rays are weakly stained by alcian blue staining. The total number of fin rays in the pectoral fin at stage 29 varies from 58 to 62 (Average = 58.4, *N*=7 skate embryos). Boxes correspond to the median and the first and third quartiles (Q1 and Q3). Whiskers extend to the last point within 1.5 times the interquartile range below and above Q1 and Q3, respectively. The scale bar is 1 mm.

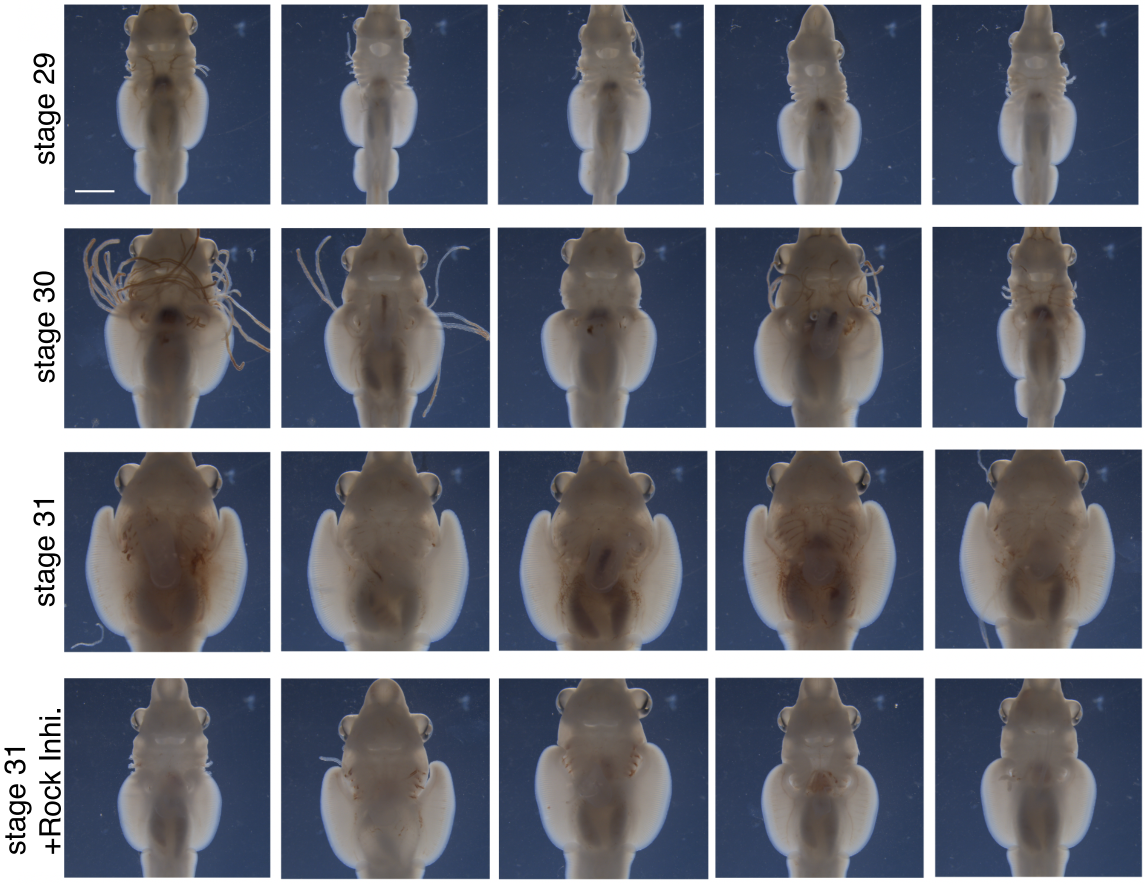

Supplementary Information Figure 10. Gross morphology of the pectoral fin in control and ROCK inhibitor-treated embryos. The pectoral fin develops to the anterior direction from stage 29 to stage 30 and to stage 31. In contrast, the pectoral fin does not grow to the anterior direction in ROCK-inhibitor treated embryos with some phenotypic variations. The external gills were removed for clear visualization of the pectoral fin in some embryos. Five replicates for each stage and condition are shown. The scale bar is 2 mm.

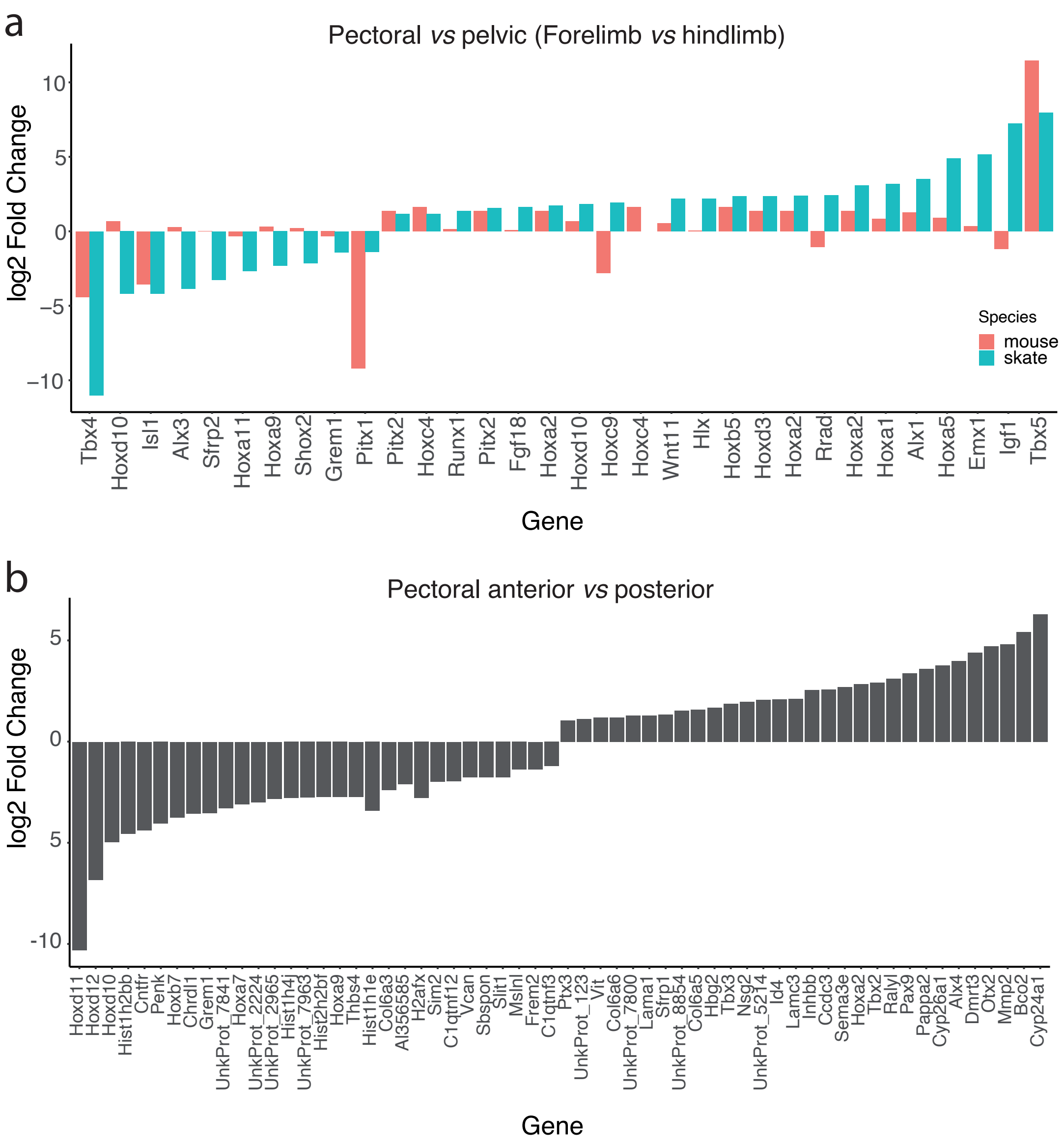

Supplementary Information Figure 11. Differential gene expression analyses a. Subset of statistically significant genes upon comparison of skate pectoral *vs*. pelvic fins RNA-seq (blue bars), in relation to the same analysis in mouse forelimb *vs*. hindlimb RNA-seq (red bars). **b.** Statistically significant genes upon comparison of anterior *vs.* posterior skate pectoral fin RNA-seq datasets.

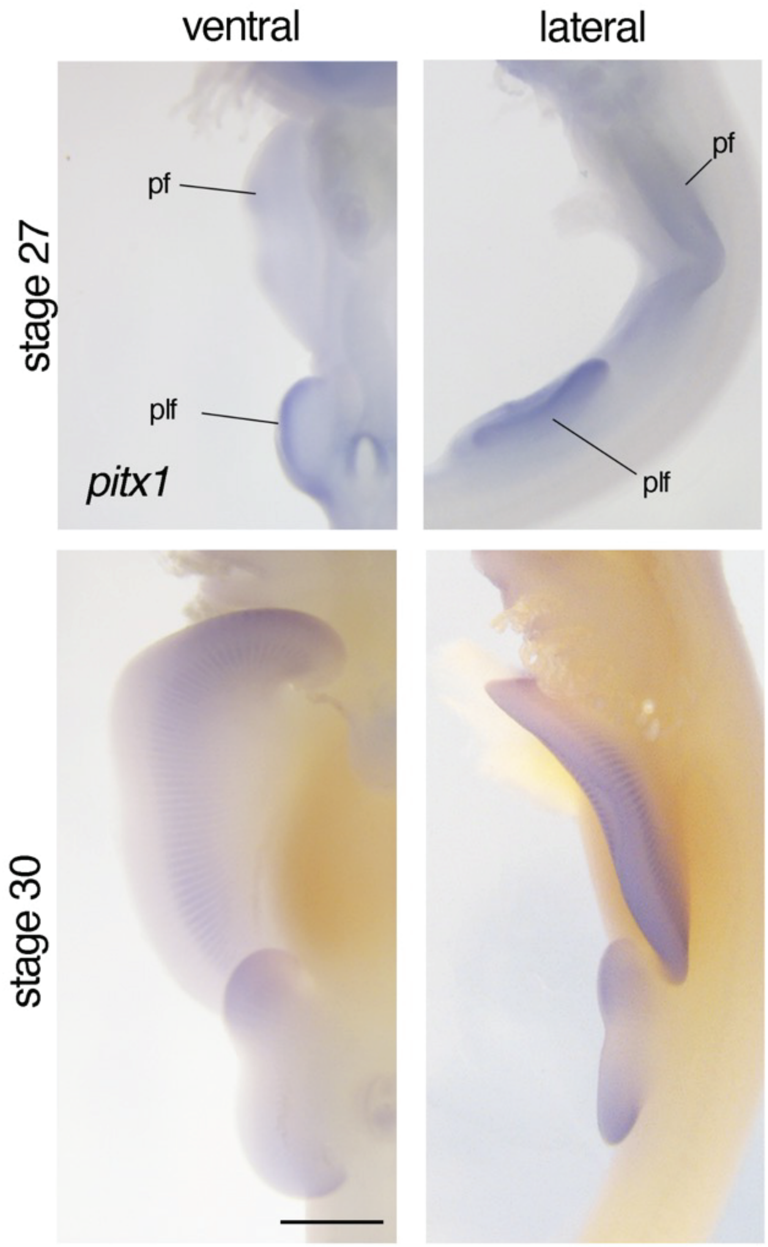

Supplementary Information Figure 12. *Pitx1* expression in skate paired fins.
Pitx1 is expressed in the pelvic fins (plf) at stage 27. At stage 30, both pectoral (pf) and pelvic fins express *pitx1*. *n* = 8 for each stage. Scale bar = 0.5 mm.

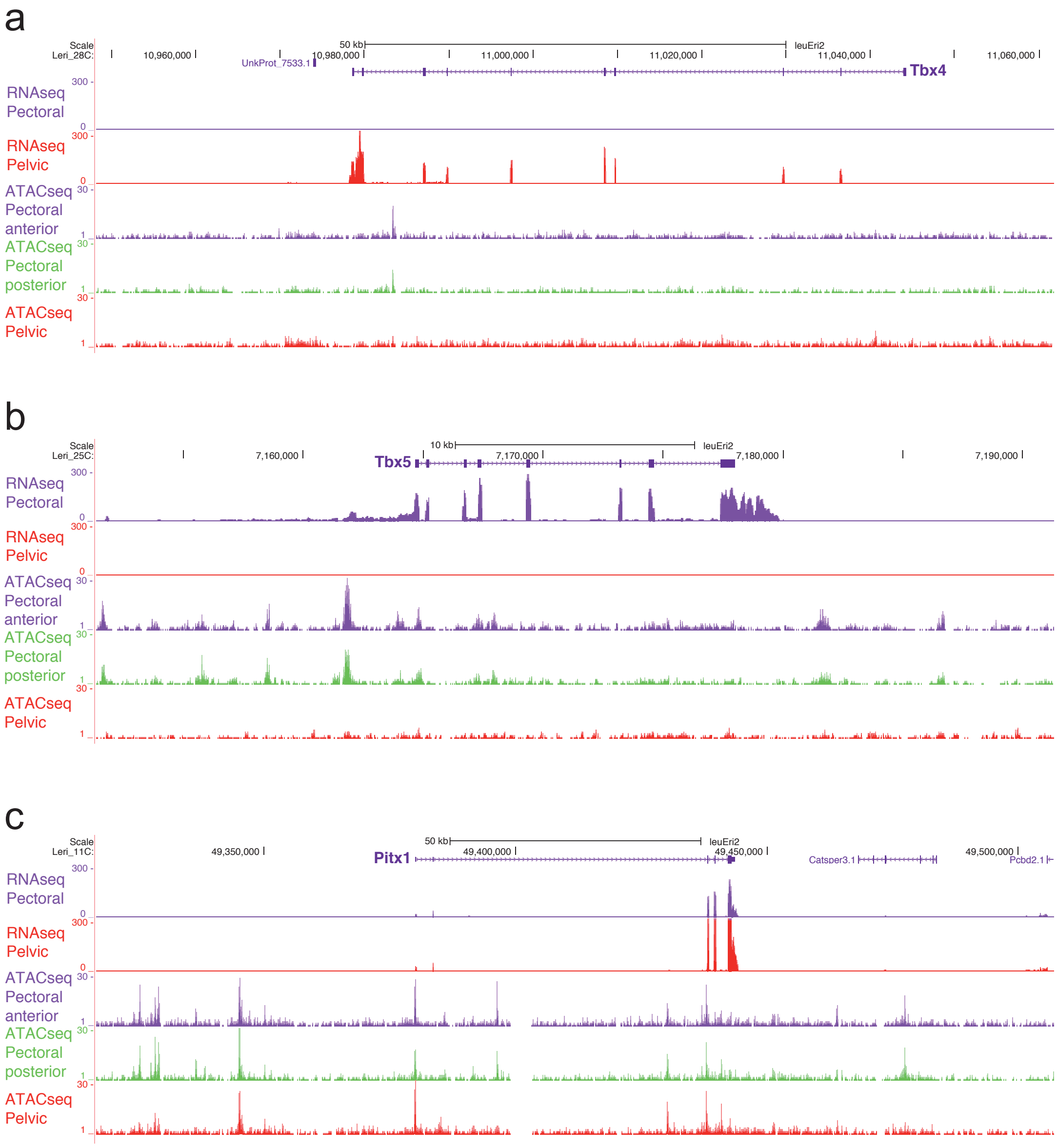

Supplementary Information Figure 13. Genomic landscape of *Tbx4*, *Tbx5* and *Pitx1*. UCSC Browser views surrounding three genes that are key for limb development and anterior-posterior identity. **a, b.** *Tbx4* and *Tbx5* are transcriptionally active in pelvic and pectoral fins, respectively, in a homologous pattern to the expression in mouse, where these genes are active in hindlimb and forelimb, respectively. **c.** Strikingly, *Pitx1* gene is expressed in both pectoral and pelvic fins, while it is only active in hindlimb in other vertebrates.

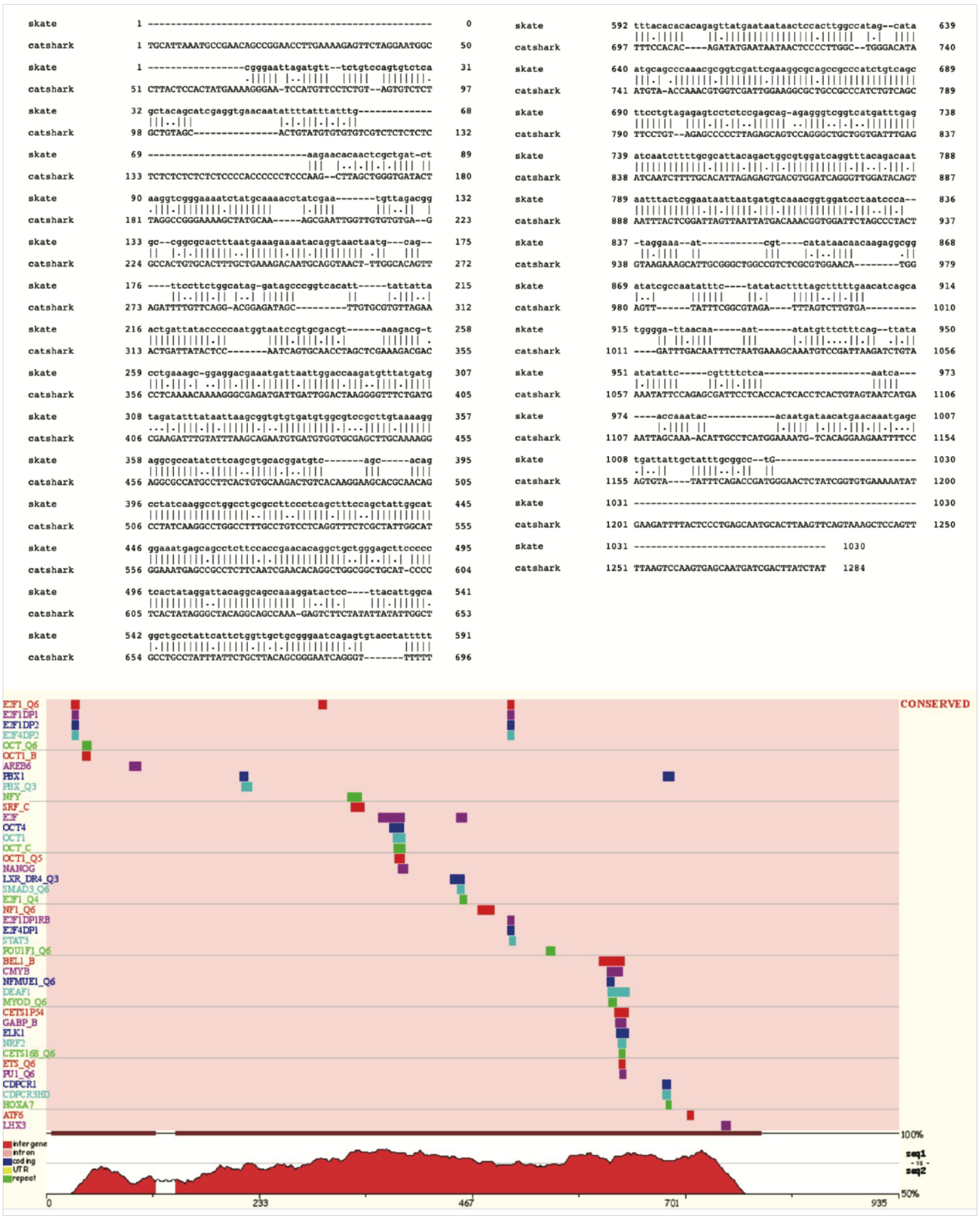

Supplementary Information Figure 14. Conservation of sequence and transcription factor binding sites between catshark and skate *HoxA* enhancers. Catshark *HoxA* enhancer (1284 bp) and skate *HoxA* enhancer (1030 bp) were aligned (top) and conserved transcription factor (TF) binding sites were identified by rVista 2.0 (bottom). Names and positions of the conserved transcription binding sites are shown by different color codes with sequence conservation peaks (red).

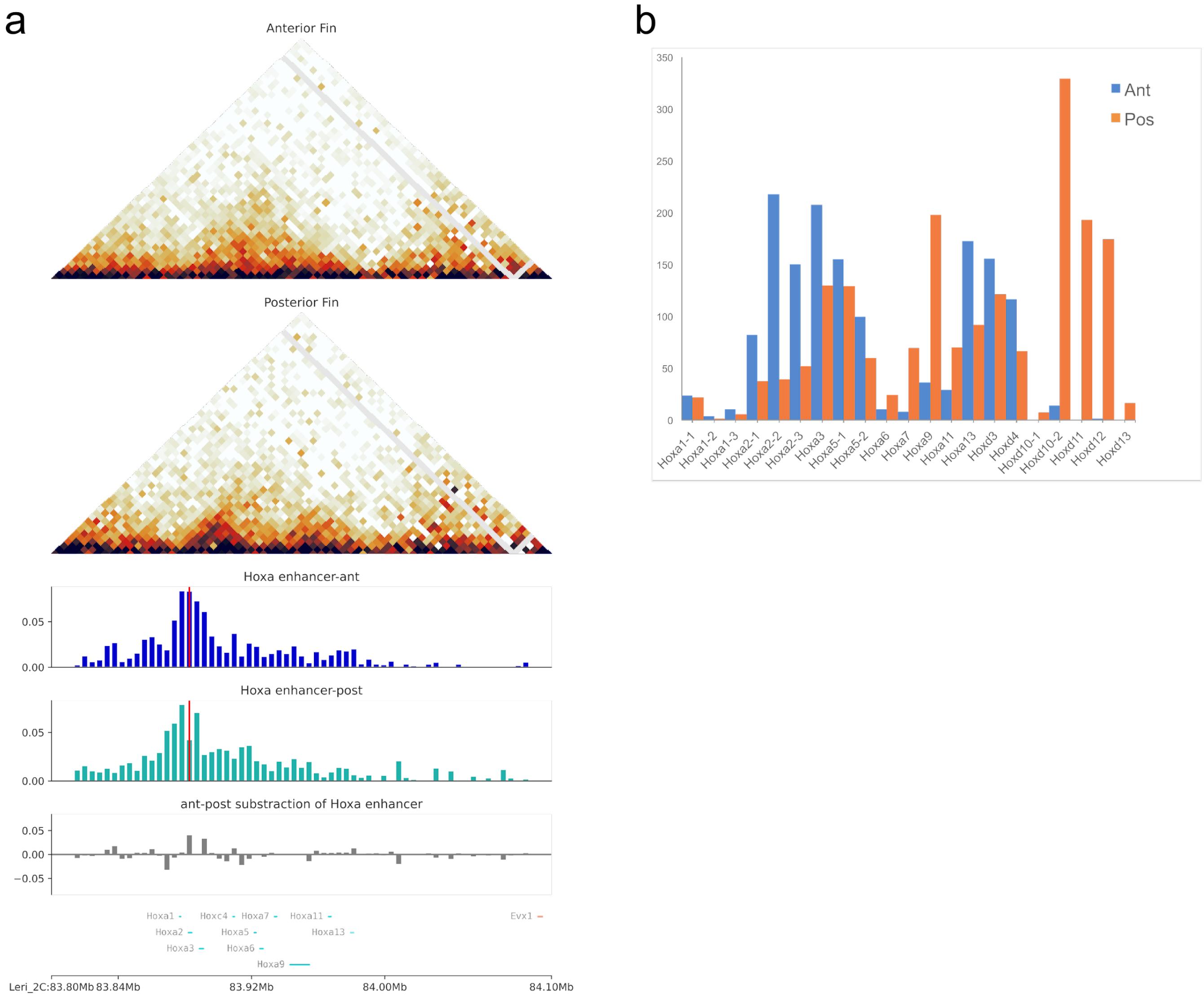

Supplementary Information Figure 15. *HoxA* enhancer contacts the whole anterior region of the *Hox* cluster and also some posterior genes. a. HiC in anterior and posterior regions of the pectoral fin at the *HoxA* cluster, and virtual 4C signal from the HiChIP data covering the same genomic region and taking the *HoxA* enhancer as a viewpoint. **b.** Expression levels of different *Hox* genes from clusters A and D in anterior (blue bars) and posterior (orange bars) regions of the pectoral fin. Y axis represents the average of reads assigned to each gene in all replicates for each condition.

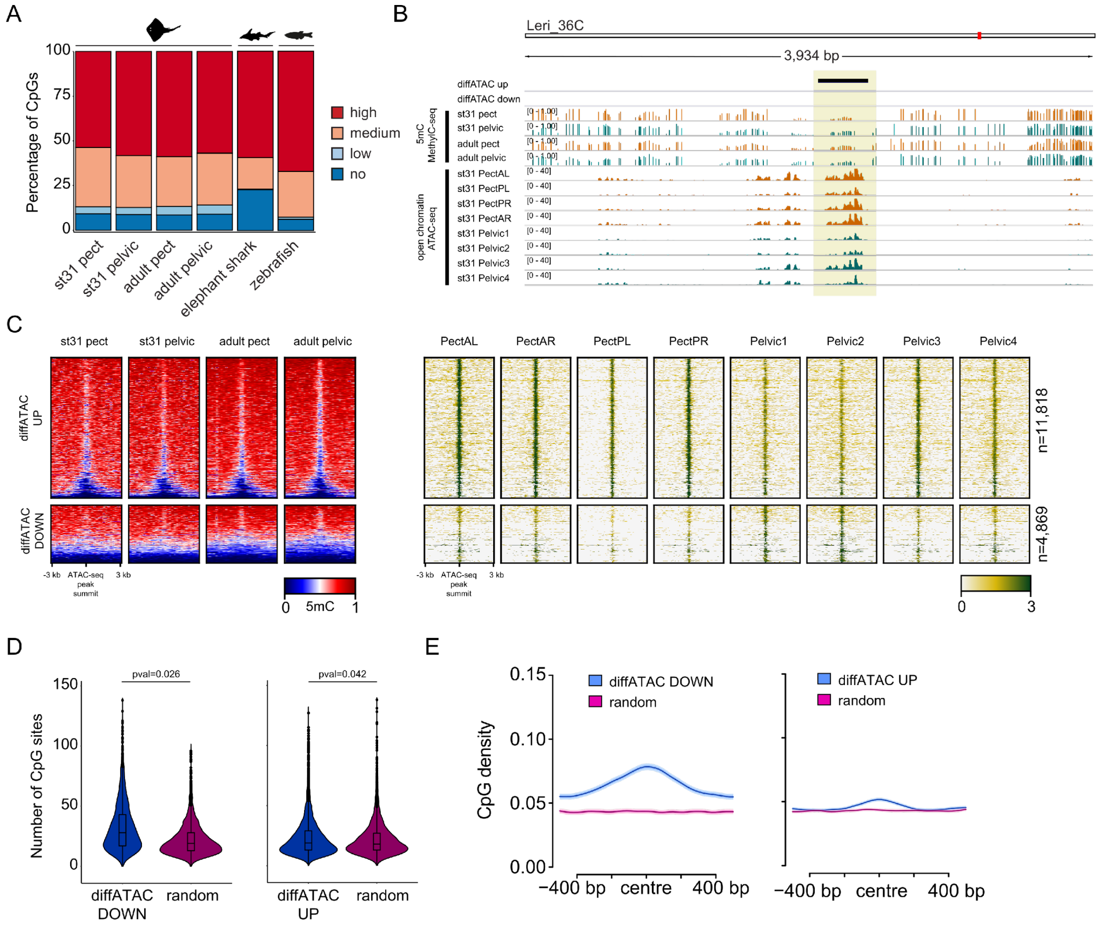

Supplementary Information Figure 16. Base resolution DNA methylome profiles of little skate embryonic and adult fins. a. Stacked bar plots indicating the percentage of methylated CpG sites in little skate (embryonic and adult pectoral and pelvic fins), elephant shark (adult liver) and zebrafish (adult liver) genomes. High, 80-100%; medium, 20-80%; low, >0-20%; no, 0% methylation. **b.** IGV browser tracks depicting DNA methylation (MethylC-seq) profiles at differentially accessible ATAC peaks between pectoral and pelvic fins. **c.** Heatmaps of DNA methylation profiles of differentially accessible ATAC peaks (UP and DOWN) in embryonic and adult pectoral and pelvic fins. Top cluster: ATAC peaks upregulated (diffATAC UP) in pectoral fins. Bottom cluster: ATAC peaks downregulated (diffATAC DOWN) in pectoral fins. **d.** Violin plots depicting the number of CpG sites within differentially accessible ATAC peaks extended +/-500 bp and 1000bp-wide random regions. Wilcoxon rank-sum test was used to compare the number of CpG sites at differentially accessible ATAC regions versus random regions. The boxes show the interquartile range (IQR) around the median; the whiskers extend from the minimum value to the maximum value unless the distance to the first and third quartiles is more than 1.5 times the IQR. One-sided Wilcoxon rank-sum test was used to compare the number of CpG sites at differentially accessible ATAC regions versus random regions **e.** Line plots depicting CpG densities of differentially accessible ATAC peaks. The number of CpG sites is calculated in 50bp windows around the ATAC peak centre or around the centre of random regions. N (diffATAC DOWN) = 4869; N (diffATAC UP) = 11818.

Supplementary Table Guide

- **SI Table 1**: Skate genome assembly statistics.
- **SI Table 2**: Genomes and proteomes used for protein clustering. Accession numbers and links for download available.
- **SI Table 3**: Fate of CLGs in skates after the WGD.
- **SI Table 4**: DEGs between pectoral and pelvic fin in skate
- **SI Table 5**: DEGs between the anterior and posterior portion of the pectoral fin in skate
- **SI Table 6**: Differentially accessible ATAC peaks between the anterior and posterior portion of the pectoral fin in skate (tsv file).
- **SI Table 7**: Differentially accessible ATAC peaks between the pectoral and pelvic fin in skate (tsv file).
- **SI Table 8**: Consensus A/B Compartment calling in skate pectoral fin Hi-C experiments.
- **SI Table 9**: Consensus TAD calling in skate pectoral fin Hi-C experiments.
- **SI Table 10**: Differential loops between anterior and posterior portions of the skate pectoral fin HiChIPs.
- **SI Table 11**: Microsyntenic pair of genes conserved between skate, garfish and mouse.
- **SI Table 12**: Genome version and lastz parameters used for global alignments prior to synteny breaks analysis.
- **SI Table 13**: Candidate genes potentially involved in rearrangements altering TADs.
- **SI Table 14**: Primers used for transgenic assays, ISH, and RT-PCRs.
