# Supplementary material for: The little skate genome and the evolutionary emergence of wing-like fins
Source: Nature. 2023 Apr 12;616(7957):495–503. doi: 10.1038/s41586-023-05868-1 (PMC10115646; doi:10.1038/s41586-023-05868-1)
Supplement: Supplementary file 2 — Reporting Summary [file 41586_2023_5868_MOESM2_ESM.pdf]

nature portfolio

Corresponding author(s): Daniel Rokhsar, Dario G. Lupiañez, Ferdinand Marletaz, Juan J Tena

Last updated by author(s): Feb 2, 2023

Reporting Summary

Nature Portfolio wishes to improve the reproducibility of the work that we publish. This form provides structure for consistency and transparency in reporting. For further information on Nature Portfolio policies, see our Editorial Policies and the Editorial Policy Checklist.

Statistics

For all statistical analyses, confirm that the following items are present in the figure legend, table legend, main text, or Methods section.

n/a	Confirmed	
☐	☒	The exact sample size (*n*) for each experimental group/condition, given as a discrete number and unit of measurement
☐	☒	A statement on whether measurements were taken from distinct samples or whether the same sample was measured repeatedly
☐	☒	The statistical test(s) used AND whether they are one- or two-sided<br>*Only common tests should be described solely by name; describe more complex techniques in the Methods section.*
☒	☐	A description of all covariates tested
☐	☒	A description of any assumptions or corrections, such as tests of normality and adjustment for multiple comparisons
☐	☒	A full description of the statistical parameters including central tendency (e.g. means) or other basic estimates (e.g. regression coefficient) AND variation (e.g. standard deviation) or associated estimates of uncertainty (e.g. confidence intervals)
☐	☒	For null hypothesis testing, the test statistic (e.g. *F*, *t*, *r*) with confidence intervals, effect sizes, degrees of freedom and *P* value noted<br>*Give P values as exact values whenever suitable.*
☒	☐	For Bayesian analysis, information on the choice of priors and Markov chain Monte Carlo settings
☒	☐	For hierarchical and complex designs, identification of the appropriate level for tests and full reporting of outcomes
☐	☒	Estimates of effect sizes (e.g. Cohen's *d*, Pearson's *r*), indicating how they were calculated

Our web collection on statistics for biologists contains articles on many of the points above.

Software and code

Policy information about availability of computer code

Data collection: No software was used for data collection

Data analysis: The following public software was used for data analysis:

- Genomescope (v. 2.0)
- Megahit (v. 1.1.1)
- dbg2olc (c. 10037fa)
- Minimap2 (v. 2.12)
- Racon (v. 1.3.1)
- BUSCO (v. 3.0.2)
- purge_haplotigs (v. 1.0.2)
- HiRise (v. 2.0.5)
- Juicebox (v. 2.1.10)
- HiGlass Browser (v. 1.11.7)
- PBjelly (PBSuite v. 15.8.24)
- STAR (v. 2.5.2b)
- stringtie (v. 1.3.3)
- TACO (v. 0.7.3)
- Trinity (v. 2.8.4)
- GMAP (v. 2018-07-04)
- Mikado (v. 1.2.1)

- Transdecoder (v. 5.5.0)
- Augustus (v. 3.3.3)
- portcullis (v 1.2.0)
- exonerate (v 2.2.0)
- Repeatmasker (v. 4.0.7)
- PASA (v. 2.5.0)
- OMA (v. 2.4.1)
- HMMer (v. 3.1b2)
- MAFFT (v. 7.3)
- BMGE (v. 1.12)
- IQTREE (v. 2.1.1)
- Phylobayes (v. 4.1e)
- Mmseq2 (v. 13-45111)
- Broccoli (v. 1.2)
- GeneRax (v. 2.0.4)
- BWA (v. 0.7.17)
- pairtools (v. 0.3.0)
- Juicer Tools (v. 1.13.02)
- FAN-C (v. 0.9.1)
- FIMO (v. 4.11.2)
- Clover (https://github.com/mcfrith/clover, 5ca3e81725)
- profileplyr (v. 1.13.0)
- HiCExplorer (v. 3.7.2)
- EdgeR (v. 3.36.0)
- TADbit (v 1.0)
- bedGraphToBigWig (kentUtils v4)
- MACS2 (v. 2.2.7)
- FitHiChIP (v. 9.0)
- Bedtools (v. 2.26.0)
- GenomicRanges (v. 1.44.0)
- Nextflow (v19.10.0)
- nf-core/rnaseq (v. 1.4)
- DeSeq2 (v. 1.30.1)
- TopGO (v. 2.42.0)
- nf-core/atacseq (v1.0.0)
- gtfToGenePred (kentUtils v4)
- lastz (v. 1.04.15)
- chainCleaner (https://github.com/hillerlab/GenomeAlignmentTools v. 971d043)
- ReactomePA (v. 1.38.0)
- ClusterProfiler (v. 4.2.2)
- Fiji (v. 20191028-2046)
- Stereomorph (v. 1.6.1)
- ShinyGM (v. 9.11.21)
- Muscle (v. 3.8.1551)
- mafft (v. 7.407)
- kalign (v. 2.04)
- T-coffee (v. 12.0)
- trimAl (v. 1.4.rev15)
- IQTREE (v. 1.6.9)
- duptree (v. 1.48)
- Trimmomatic (v. 0.32)
- WALT (v. 1.01)
- Samtools (v. 1.3)
- Picard (v. 2.3.0)
- MethylDackel (v. 0.6.1)
- deepTools (v. 3.5.0)

Custom code is available at:

https://gitlab.com/skategenome
hic_pipe.py / filt2hic.sh (https://gitlab.com/rdacemel/hic_ctcf-null)

For manuscripts utilizing custom algorithms or software that are central to the research but not yet described in published literature, software must be made available to editors and reviewers. We strongly encourage code deposition in a community repository (e.g. GitHub). See the Nature Portfolio guidelines for submitting code & software for further information.

Data

Policy information about availability of data

All manuscripts must include a data availability statement. This statement should provide the following information, where applicable:

- Accession codes, unique identifiers, or web links for publicly available datasets
- A description of any restrictions on data availability
- For clinical datasets or third party data, please ensure that the statement adheres to our policy

Raw and processed sequencing data were deposited in GEO (GSE188980 and GSE190730) and SRA (PRJNA783899). Mouse hindlimb RNA-seq data used for comparative analyses are publicly available under GEO accession number GSE104459 and mouse forelimb RNA-seq data under GEO accession number GSE136437. Zebrafish and elephant shark bisulfite sequencing data used for comparison were downloaded from PRJNA379367 and GSE122723136 accession codes respectively. Skate anterior and posterior pectoral fins RNA-seq data are publicly available under BioProject accession code PRJNA288370.

Human research participants

Policy information about studies involving human research participants and Sex and Gender in Research.

Reporting on sex and gender	*Use the terms sex (biological attribute) and gender (shaped by social and cultural circumstances) carefully in order to avoid confusing both terms. Indicate if findings apply to only one sex or gender; describe whether sex and gender were considered in study design whether sex and/or gender was determined based on self-reporting or assigned and methods used. Provide in the source data disaggregated sex and gender data where this information has been collected, and consent has been obtained for sharing of individual-level data; provide overall numbers in this Reporting Summary. Please state if this information has not been collected. Report sex- and gender-based analyses where performed, justify reasons for lack of sex- and gender-based analysis.*
Population characteristics	*Describe the covariate-relevant population characteristics of the human research participants (e.g. age, genotypic information, past and current diagnosis and treatment categories). If you filled out the behavioural & social sciences study design questions and have nothing to add here, write "See above."*
Recruitment	*Describe how participants were recruited. Outline any potential self-selection bias or other biases that may be present and how these are likely to impact results.*
Ethics oversight	*Identify the organization(s) that approved the study protocol.*

Note that full information on the approval of the study protocol must also be provided in the manuscript.

Field-specific reporting

Please select the one below that is the best fit for your research. If you are not sure, read the appropriate sections before making your selection.

☒ Life sciences ☐ Behavioural & social sciences ☐ Ecological, evolutionary & environmental sciences

For a reference copy of the document with all sections, see nature.com/documents/nr-reporting-summary-flat.pdf

Life sciences study design

All studies must disclose on these points even when the disclosure is negative.

Sample size	Two replicates were used for Hi-C experiments as it is widely recommended in the field (see recent Rao et al. 2017, Cell; Franke et al. 2020, Nature Communications; Valton et al. 2022, Nature Structural & Molecular Biology). Two replicates were also used for HiChIP (Franke et al. 2020) and ATAC-seq (see Marlétaz et al. 2017, Nature) following a similar rationale. Three different catshark embryo pectoral fins were used for RT PCR detection of Prickle1 as it is widely accepted to devise robust statistics. Five replicates were used for the whole mount ISH as it is widely accepted in the field of cartilaginous fish research (see Marconi et al. eLife 2020). For enhancer testing in zebrafish, in F0, GFP positive embryos were selected after transgenesis mix injection, and the ratio of GFP fin positive vs. GFP fin negative embryos was calculated (5/18 for the skate enhancer, 0/31 for the shark enhancer). Regarding the F1 stable transgenic lines, they were considered as stable lines when three independent founders with the same GFP expression pattern were found. For the validated Hox overexpression line 57 embryos for the control experiment and 93 embryos for the Dexamethasone treatment were used, which is in the order of previously used in other similar studies (Tena et al., Dev. Biol. 2007 301:518-31; Freitas et al. Dev. Cell 2012 23:1219-29). These numbers guarantee the robustness and reliability of statistical analyses. For the cell elongation analyses, about 400-550 cells were analyzed in each domain of the pectoral fin (the precise numbers are in the figure caption) as 50-200 cells are typically analyzed as replicates in PCP analysis (see Butler and Wallingford 2018). These numbers were enough to show statistically significant differences among samples. Five replicates were used for the whole body inhibitor treatment as it is widely accepted in the field of<br>cartilaginous fish research (see Marconi et al. eLife 2020, for example). For the beads implantation experiments, we prepared 9-10 replicates for each condition as the locations of the implantation may vary due to the manual surgery. Despite a certain amount of variation, the results showed an obvious difference with/without the inhibitor (6/10 embryos vs 0/10 embryos showed aberrant fins, respectively). For bisulfite sequencing, as per Burger et al NAR 2013, we used single replicates of skate MethylC-seq data (at coverage > 10X), to identify UMRs and

	LMRs.
Data exclusions	An experiment with two replicates was originally designed for the HiChIPs of the anterior portion of the pectoral fin, but one of the replicates' quality was not high enough and we had to discard it. Given the reliability of the technique and the redundancy and overlapping with the newly presented high-resolution HiC data in anterior and posterior pectoral fins, we decided not to repeat this lost replicate.
Replication	All experiments were performed at least in two replicates, refer to sample size section for details.
Randomization	Randomization was not directly employed since we were analyzing developmental processes and phenotypes in skate, catshark and zebrafish embryos. No external covariates are expected and since embryos are collected from many different parental individuals, randomization strategies are not obvious and they are not considered necessary with animal models like skates and zebrafish.
Blinding	Blinding was not relevant for our study since all comparisons were performed automatically using statistical software not influenced by the investigator.

Reporting for specific materials, systems and methods

We require information from authors about some types of materials, experimental systems and methods used in many studies. Here, indicate whether each material, system or method listed is relevant to your study. If you are not sure if a list item applies to your research, read the appropriate section before selecting a response.

Materials & experimental systems

n/a	Involved in the study
☐	☒ Antibodies
☒	☐ Eukaryotic cell lines
☒	☐ Palaeontology and archaeology
☐	☒ Animals and other organisms
☒	☐ Clinical data
☒	☐ Dual use research of concern

Methods

n/a	Involved in the study
☒	☐ ChIP-seq
☒	☐ Flow cytometry
☒	☐ MRI-based neuroimaging

Antibodies

Antibodies used	We have used anti-Histone H3 (tri methyl K4) antibody - ChIP Grade from Abcam (ab8580) for the HiChIPs experiments
Validation	This antibody has been tested by the commercial company in experiments of chromatin immunoprecipitation, among others, in cow and human, but it has been predicted to work in many other species of vertebrates, invertebrates and even plants. In our lab we have successfully performed ChIP-seq and HiChIP experiments with this antibody in several species like zebrafish, amphioxus or sea urchin.

Animals and other research organisms

Policy information about studies involving animals; ARRIVE guidelines recommended for reporting animal research, and Sex and Gender in Research

Laboratory animals	This study did not require the use of adult animals. Skate and elephant shark (Scyliorhinus retifer) embryos were obtained from the Marine Resource Center at Marine Biological Laboratory (MA, USA) and were used for each experiment at Marine Biological Laboratory and Rutgers University. Zebrafish embryos were obtained from AB and Tübingen strains at the fish facility of Centro Andaluz de Biología de Desarrollo (Seville, Spain). Sex was not determined since it is unfeasible for the embryos analyzed. Developmental stages are always stated throughout the manuscript and figure captions.
Wild animals	The study did not involve wild animals
Reporting on sex	Sex was not determined since it is unfeasible for the embryos analyzed. Developmental stages are always stated throughout the manuscript and figure captions.
Field-collected samples	The study did not involve field-collected samples.
Ethics oversight	Experiments with skate embryos were performed at the Marine Biological Laboratory and Rutgers University under these protocols: MBL IACUC protocol #18-36 and Rutgers IACUC protocol #201702646. Zebrafish procedures were reviewed and approved by the Ethical Committees from the University Pablo de Olavide, CSIC, and the Andalusian government, and performed in compliance with all relevant ethical regulations.

Note that full information on the approval of the study protocol must also be provided in the manuscript.
